# Dihedral Angle Measurements for Structure Determination by Biomolecular Solid-State NMR Spectroscopy

**DOI:** 10.3389/fmolb.2021.791090

**Published:** 2021-12-06

**Authors:** Patrick C. A. van der Wel

**Affiliations:** Solid-state NMR Group, Zernike Institute for Advanced Materials, University of Groningen, Groningen, Netherlands

**Keywords:** solid-state NMR, structural biology, amyloid, polyglutamine, protein structures

## Abstract

In structural studies of immobilized, aggregated and self-assembled biomolecules, solid-state NMR (ssNMR) spectroscopy can provide valuable high-resolution structural information. Among the structural restraints provided by magic angle spinning (MAS) ssNMR the canonical focus is on inter-atomic distance measurements. In the current review, we examine the utility of ssNMR measurements of angular constraints, as a complement to distance-based structure determination. The focus is on direct measurements of angular restraints via the judicious recoupling of multiple anisotropic ssNMR parameters, such as dipolar couplings and chemical shift anisotropies. Recent applications are highlighted, with a focus on studies of nanocrystalline polypeptides, aggregated peptides and proteins, receptor-substrate interactions, and small molecule interactions with amyloid protein fibrils. The review also examines considerations of when and where ssNMR torsion angle experiments are (most) effective, and discusses challenges and opportunities for future applications.

## Introduction

In modern integrative structural biology, complementary structure determination methods provide insights into different aspects of the structure, dynamics and co-assembly of biomolecules. Tremendous advances in solid-state NMR (ssNMR) and in particular magic-angle-spinning (MAS) NMR spectroscopy have widened the contribution of this technique to our understanding of protein aggregates and assemblies (Van der Wel, 2018). Descriptions of the process of structure determination via ssNMR spectroscopy often focus on the role of inter-atomic (or rather inter-nuclear) distances. However, NMR-based insights into the local geometry of protein backbones, in the form of dihedral angles, can also be a powerful tool in the NMR-based structural biology arsenal. In this review article we examine MAS ssNMR techniques that allow direct insights into angular constraints that define the structures of proteins and other biomolecules. Readers are also referred to earlier reviews in the mid 2000s (Hong, 2006; Hong and Wi, 2006; Ladizhansky, 2009), and the current review will focus on more recent applications since 2006. We shall also examine the place of these techniques in current and future biomolecular MAS ssNMR. Examples show how these restraints can be effective and even essential tools for structure determination of specific kinds of biological structures and assemblies.

## Torsion Angles in Proteins

**FIGURE 1:**
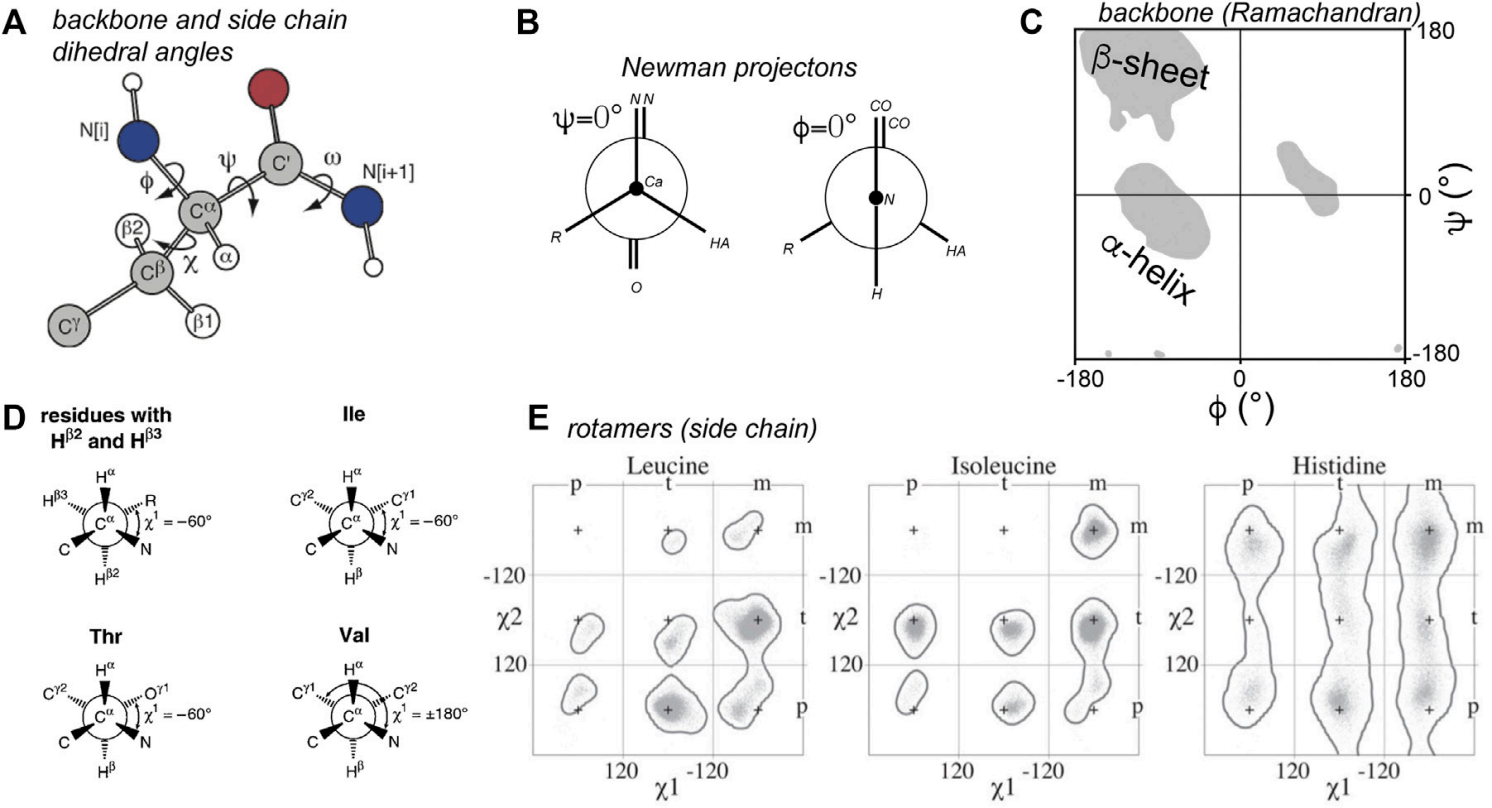
Torsion angles in proteins. **(A)** Dihedral angles defining the protein backbone and side chain: ϕ, ψ, and ω, and χ. **(B)** Newman projections of the ϕ and ψ angles. The zero degree configuration of each is shown. **(C)** Ramachandran plot of ϕ and ψ angles. Grey areas indicate the classic secondary structure regions that are most populated. Note that the ϕ = 0° from panel B is not usually observed due to steric hindrance. **(D)** Definitions of the χ_1_ side chain angle. **(E)** Rotamer states for χ_1_ and χ_2_ for three residue types. Panel A is adapted with permission from (Hong et al., 1997a), **(D)** is adapted with permission from (Markley et al., 1998), and panel E from (Hintze et al., 2016).

By these dihedral or torsion angles we refer to the angle of two neighboring chemical bonds with each other (Figure 1). In protein structural biology the torsion angles that define the backbones of proteins are most commonly discussed, and they have a standard nomenclature (IUPAC-IUB Commission on Biochemical Nomenclature., 1970; Markley et al., 1998). These angles are defined and illustrated in Figures 1A,B. Among these the ω angle is not very variable, with it typically adopting a value of ∼180° (trans). The cis configuration is rare, but when it occurs it can have notable biological consequences, for instance Pro cis/trans isomerization is implicated in the aggregation process of the β2-microglobulin protein (Torbeev and Hilvert, 2013; Mukhopadhyay et al., 2018). Of more common interest are the *ϕ* and *ψ* backbone angles, which are visualized in Ramachandran plots (Figure 1C) (Ramachandran and Sasisekharan, 1968; Hovmöller et al., 2002). Only particular regions of Ramachandran space tend to be accessible, with the highly populated clusters representing secondary structure elements. The side chains of amino acids are also defined by named torsion angles, called *χ*_1_, *χ*_2_ etc., following an accepted nomenclature (Figures 1D,E) (IUPAC-IUB Commission on Biochemical Nomenclature., 1970; Markley et al., 1998; Lovell et al., 2000). These angles have preferred regions of geometric space, known as the preferred rotamer states that are nicely captured in rotamer libraries of evolving sophistication (Figures 1E,F) (Lovell et al., 2000; Dunbrack, 2002; Hintze et al., 2016).

## SSNMR Torsion Angle Measurements—NCCN as an Example

### Technical Implementation and Analysis

The general principle of torsion angle measurements by ssNMR is based on the measurement of the relative orientations of anisotropic ssNMR parameters, which in turn can be correlated to bond angles or orientations. The most straightforward and most common example involves the relative orientations of two different dipolar couplings, since these dipolar interactions are conveniently aligned with chemical bonds. For example, one can measure the relative orientation of the dipolar coupling vector (between ^13^C and ^15^N) of one C-N bond to the dipolar vector associated with a second C-N bond. When these two vectors are associated with the Cα-N and C′-N bonds in a polypeptide, the angle would be equivalent to the ψ angle (Figure 1A). This is the approach behind the “NCCN” torsion angle experiments introduced in the late 1990s (Feng et al., 1997a; Costa et al., 1997).

**FIGURE 2:**
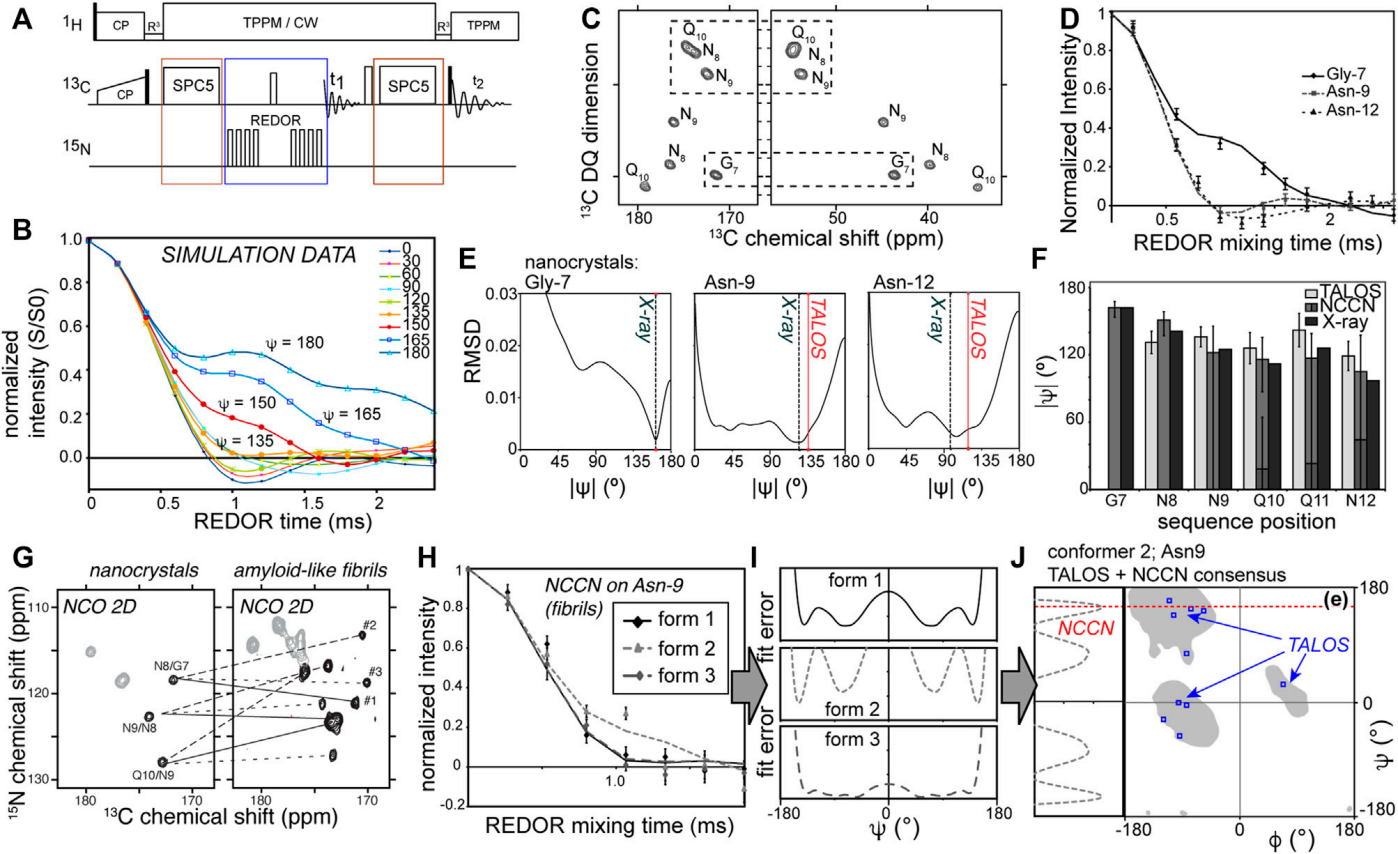
Measuring backbone angle ψ via NCCN experiments. **(A)** Pulse sequence for a 2D NCCN experiment, in which 2D ^13^C-^13^C (SQ-DQ) spectra are acquired for varying lengths of ^13^C-^15^N dipolar mixing times. **(B)** Simulated NCCN dephasing curves, showing the S/S0 intensity ratios as a function of the REDOR mix time. **(C)** Example 2D SQ-DQ spectrum acquired as part of a NCCN measurement of amyloid-like peptide nanocrystals, along with **(D)** experimental NCCN curves for three residues. **(E)** RMSD fit results of the experimental data, showing the minima. The X-ray and TALOS results are marked as well. **(F)** Bar graph of the NCCN best-fit results compared to the TALOS chemical shift analysis and the known X-ray structure data. The NCCN data match the X-ray data, with a better accuracy than TALOS analysis alone. **(G)** 2D NCO spectra for nanocrystalline and fibrillar GNNQQNY peptide (uniformly ^13^C,^15^N-labeled in the first four residues, marked as residues G7 through Q10 of the parent Sup35p protein). Lines show how each residue gives one signal in the crystals, but three peaks in the fibrils, which complicates distance measurements. **(H)** A NCCN experiment results probing the backbone angle ψ for Asn-9 in the three co-existing fibril conformers. Two conformers have similar dephasing curves, but conformer #2 diverges. **(I)** RMSD curves between simulation and experiment for the full range of ψ angles, for each conformer. Note that the minima occur at multiple angles, and sometimes are broad, providing ambiguous constraints. **(J)** To resolve this one can combine chemical shift analysis (TALOS) with the NCCN constraints. In this example the 10 best TALOS “solutions” are not self-consistent, but by considering the NCCN angle, we pinpoint the optimal result in the β-sheet region near ψ = 150°. Panels C-J are reprinted with permission from ref. (van der Wel et al., 2010) copyright 2010 American Chemical Society.

The original implementation of NCCN measurements (Rienstra et al., 2002a, 2002b; Ladizhansky et al., 2003; Jaroniec et al., 2004; Bajaj et al., 2009; Barnes et al., 2009; van der Wel et al., 2010; Hoop et al., 2016) is as follows: a double quantum (DQ) state is generated between the directly bonded Cα and C′ carbons, e.g., via SPC5 dipolar recoupling (Figure 2A; red boxes). This DQ state is then submitted to ^15^N-^13^C recoupling (commonly via the REDOR approach; Figure 2A, blue box). Since each carbon has a directly attached ^15^N, they both experience rapid dephasing in a manner that is dominated by the directly-bonded ^15^N. Whilst the orientation dependence of a single ^15^N-^13^C REDOR recoupling experiment is masked in a typical MAS ssNMR study, these two ^15^N-^13^C recoupling curves display an interference effect that results in behavior that is sensitive to the N-Cα-C′-N dihedral angle (Figure 2B).

Practically speaking, a typical REDOR approach is used to measure a relaxation-corrected dephasing curve (Figure 2B). A series of datasets with varying REDOR mixing times is acquired (keeping the DQ excitation time fixed). The relaxation correction involves the measurement (at each REDOR time) for each peak of interest of a “S” signal (*with* active ^15^N REDOR pulses) and “S0” signal (without ^15^N REDOR pulses). The S/S0 peak intensity ratio is then plotted, yielding angle-dependent curves as shown in Figure 2B. At each time point either a 1D or 2D spectrum is acquired, with the 2D implementation shown in Figures 2A,C (Ladizhansky et al., 2003; van der Wel et al., 2010). The main benefit of the 2D version is that it allows one to resolve many signals at once (Ladizhansky et al., 2003). Naturally, it comes at the expense of signal-to-noise (per unit time), which is superior in the 1D versions. It is worth noting that the signal-to-noise can be a challenge in these experiments. This stems from the fact that a clear distinction of the different dihedral angles only occurs in those time points where already extensive dephasing has occurred (i.e., much of the signal is lost; see Figure 2B). Moreover, in this region, the differences between some of the dihedral angles can be quite small, such that a very good signal to noise may be required to narrow down a specific angle. Achieving a good signal-to-noise level is inherently a challenge due to the polarization losses associated with the multiple recoupling steps (both the ^13^C DQ recoupling and filtering, and also the heteronuclear recoupling), both due to losses inherent in the DQ filtering and relaxation processes.

Analysis and interpretation of the obtained data is done by comparing experimental data points (i.e., the S/S0 ratios) to reference curves such as the data in Figure 2B. The latter can be derived from analytical descriptions of the experiment. In our own analyses we typically employ primarily numerical simulations to generate the curves. A relevant spin system is modelled for the range of candidate dihedral angles (or molecular structures), resulting in a library of reference curves. For some types of torsion angle experiments it may also be necessary to incorporate in this “library” relaxation effects, the impact of the chemical shift anisotropy (CSA), and dynamic modulation (van der Wel et al., 2009; Hou et al., 2010; Hoop et al., 2014). Naturally, these additional free parameters increase the complexity of the analysis and reduce the expected precision of obtained results.

**FIGURE 3:**
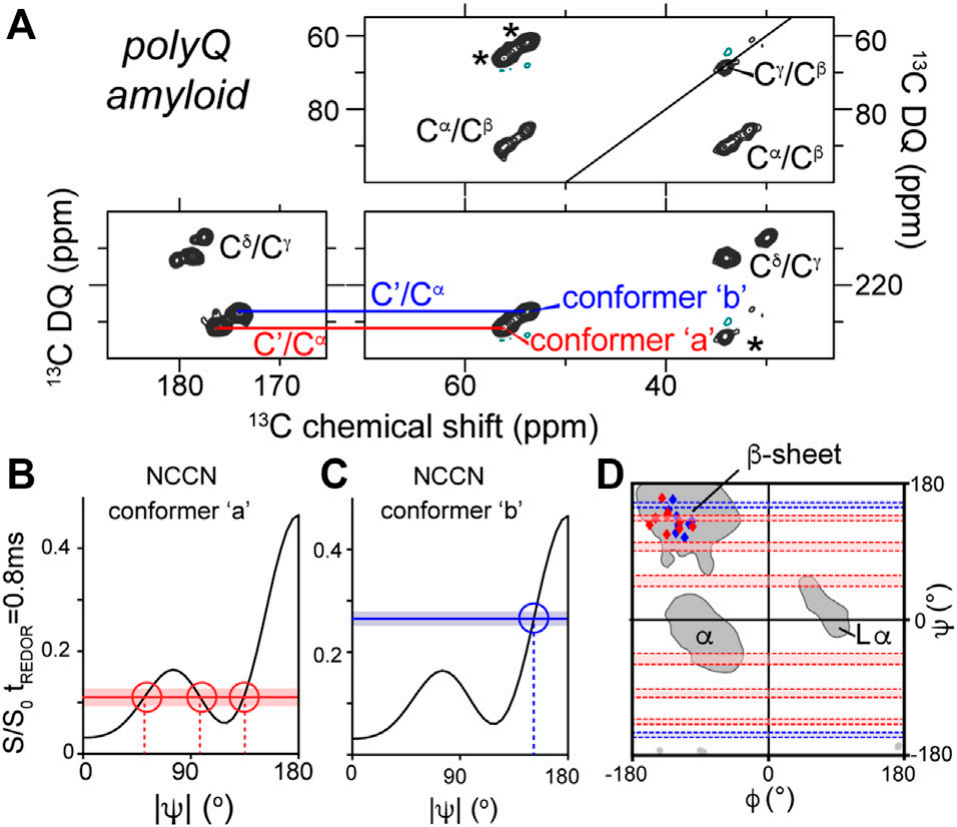
NCCN analysis of polyQ amyloid structure. **(A)** 2D SQ-DQ spectrum from a NCCN measurement on polyQ amyloid fibrils, showing the main peaks of the two conformers called ‘a’ (red) and ‘b’ (blue) that make up the polyQ amyloid core. **(B,C)** Comparison of experimental S/S_0_ values (horizontal lines) to simulated ψ-dependence S/S_0_ ratios (black lines) for each conformer. **(D)** Ramachandran plot showing the integration of TALOS and NCCN analysis of the structures of polyQ amyloid conformers a and b. See also Figure 4 below. Figure adapted with permission from ref. (Hoop et al., 2016).

Additionally, it is worth noting that a dipolar recoupling experiment like REDOR reflects a through-space interaction and can therefore invoke multispin interactions in extensively or fully isotope-labeled samples. Such multispin interactions would not invalidate the dihedral angle experiment, as it is essentially based on seeing a dependence of the observed signal on the dihedral angle in question. This dependency would be modulated, but not be negated by multispin interactions, unless non-local interactions would dominate over the local interactions which are supposed to be recoupled. However, in this case the effect of “dipolar truncation”, in which strong dipolar interactions suppress or truncate the contributions from longer range interactions (Bayro et al., 2009), is actually beneficial. Directly-bonded ^13^C-^15^N interactions will effectively truncate the effect of long-range inter-residue and inter-molecular interactions. It is also worth noting that we benefit here from the relatively low density of ^15^N sites in polypeptides, which allows for the trains of ^15^N REDOR pulses without detrimental ^15^N-^15^N recoupling (illustrated in Figure 2A). Another important observation is that these experiments can be applied to polypeptides outfitted with uniform ^13^C and ^15^N labeling, without need for site- or residue-specific isotope labeling as may be needed for certain other types of torsion angle experiments. The examples in Figures 2, 3 are obtained with synthetic peptides where short stretches of residues were labeled, but the same experiments can be applied to fully labeled proteins (Ladizhansky et al., 2003).

### Example Application to Nanocrystalline Peptides With Known Structures

The data in Figures 2C–F represent validation experiments in an amyloid-like, but nanocrystalline, peptide assembly. Integrated peak volumes from the SQ-DQ 2D spectrum (Figure 2C) are plotted as S/S0 ratios as a function of the REDOR recoupling time. The example curves in Figure 2D show the variable differences between residues, with some of the angles hard to distinguish (see also Figure 2B for ψ < 130°). Figure 2E shows the results of fitting the experimental curves, yielding in some cases multiple minima. This illustrates one common challenge of torsion angle experiments, which is that they often have regions of angular space where the obtained dephasing curves are essentially indistinguishable. As reviewed in earlier work (Hong, 2006; Hong and Wi, 2006; Ladizhansky, 2009), this issue can be addressed by combining multiple types of dihedral angle measurements with complementary sensitivities. Notably, the NCCN experiment minima are close to both the X-ray structure angles and the results of NCCN-based chemical shift analysis. This is illustrated in the bar graphs of Figure 2F. These results show that the NCCN experiment gives results that match the structure as known from X-ray microcrystallography (Nelson et al., 2005; van der Wel et al., 2007, 2010), consistent with earlier studies (Ladizhansky et al., 2003).

### Studies of Unknown Structures in Amyloid Fibrils

Several studies have deployed NCCN experiments to study unknown structures of amyloid-like fibrils (Jaroniec et al., 2004; van der Wel et al., 2010; Hoop et al., 2016). For the fibrils formed by the abovementioned prion-derived model peptide GNNQQNY, these experiments detected the anticipated β-sheet structures typical of amyloids, but also non-β structure as an integral part of a composite fibril structure (van der Wel et al., 2010; Lewandowski et al., 2011). Notably this feature was present in one of three conformers (monomer structures) that composed the “composite” fiber architecture (which manifest as three peaks per residue; Figure 2G). This feature made the reliance on distance measurements more difficult, due to substantial peak overlap. Thus, the complementary torsion angle measurements were particularly helpful and valuable. The three conformers’ distinct structures are clear in the torsion angle data, as illustrated for residue Asn-9 in Figure 2H. The presence of a non-β kink or turn was at first surprising, as it is absent from the nanocrystals. However, nowadays this finding is reminiscent of the common presence of kinks, turns or bends in amyloid architectures (between quite short β-strand segments) (van der Wel, 2017; Sawaya et al., 2021). Figures 2I,J shows how the experimental ssNMR data were translated to dihedral angles. The fit between the experimental data and simulated curves was evaluated as a function of the simulated dihedral angle (Figure 2I). In some cases this shows consistency (i.e., low RMSD between the experimental data and simulated data) with multiple possible angles. One approach to overcome this ambiguity, shown in Figure 2J, is to combine the optimal NCCN angles with TALOS-based chemical shift analysis, in order to obtain a unique solution not accessible through either approach alone.

A similar structural complexity was subsequently detected in ssNMR studies of polyglutamine (polyQ) amyloid structure, which was found to consistently contain two distinct types of peptide conformations (Figure 3A) (Schneider et al., 2011; Sivanandam et al., 2011; Hoop et al., 2014, Hoop et al., 2016; Isas et al., 2015; Matlahov and van der Wel, 2019). Proteins with expanded polyQ domains are of biomedical interest as they represent the molecular basis of a series of CAG repeat expansion disorders, which remain as-yet incurable and untreatable (Wetzel, 2012). The disease-associated mutant proteins are prone to aggregation and form toxic aggregates, which include fibrillar structures with all the hallmarks of typical amyloid fibrils. Unlike several other amyloid proteins (van der Wel, 2017; Sawaya et al., 2021), the 3D atomic structure of none of the pathogenic polyQ protein aggregates is as yet known (Matlahov and van der Wel, 2019). The degenerate nature of the primary sequence of these polypeptides greatly complicates structural analysis by ssNMR, which has made the deployment of torsion angle measurements valuable and indeed essential. Structural study by distance measurements is further complicated by the already noted composite nature of the amyloid core structure, presenting as two peaks for each residue (marked “a” and “b” in Figure 3). By combining NCCN measurements and TALOS-based chemical shift analysis, it was shown that these two conformers reflect two types of β-strand structures with distinct backbone conformations (Figures 3A–D) (Hoop et al., 2016). The origin of this characteristic doubled-peaks signature stems from the presence of antiparallel β-sheets in these fibrils, which contain β-hairpin motifs for the longer polyQ expansion lengths (more about polyQ structures below) (Matlahov and van der Wel, 2019).

## Beyond the Protein Backbone—HCCH Experiments

**FIGURE 4:**
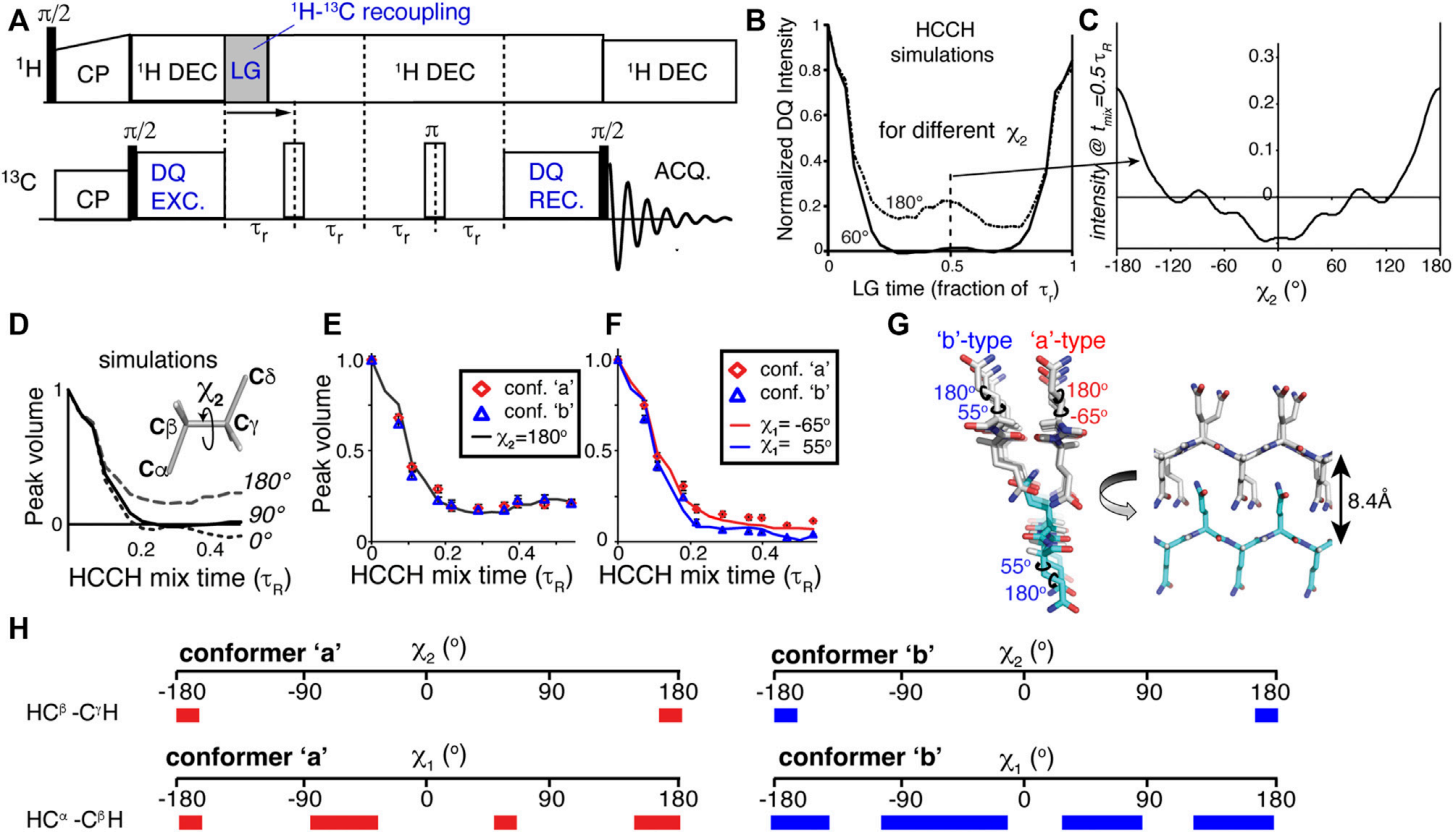
HCCH experiments for measuring side chain angles. **(A)** Pulse program schematic for a HCCH experiment. **(B)** Simulated HCCH curves for measurement of the χ_2_ angle in glutamine residues (a CβH_2_-CγH_2_ spin system), for χ_2_ = 60 or 180°. **(C)** Variation of the mid-rotor-period intensity as a function of the χ_2_ angle. **(D,E)** Simulated and experimental data measuring the χ_2_ angle of glutamine residues in the core of polyglutamine amyloid. **(F)** Application to the χ_1_ angle in polyglutamine amyloid. **(G)** Visualized structural model for how the polyglutamine amyloid core is structured, based on the best-fit dihedral angle solutions. **(H)** Overview of the ambiguity in some, but not all, of the measured χ angles. The colored bars indicate those angles that are consistent with the observed torsion angle curves. The red bars on the left apply to conformer ‘a’, and the blue ones (right) to conformer ‘b’. Figure adapted from ref. (Hoop et al., 2016) with permission.

The first examples of direct MAS ssNMR torsion angle measurements were the HCCH experiments (Feng et al., 1996; Feng et al., 1997b), whose principles were also discussed in some detail in prior reviews (Hong and Wi, 2006; Ladizhansky, 2009). Briefly, again a DQ state is generated between two directly bonded ^13^C carbons, but now it is combined with controlled recoupling of the C-H dipolar interaction (Figure 4A). Keeping the DQ excitation time fixed, the C-H recoupling time is varied. This yields a time-dependent decrease in the DQ signal, with the shape of the curve dependent on the geometry (i.e., torsion angle) of the HC-CH spin system (Figure 4B) (Feng et al., 1997b; Bajaj et al., 2009; Edwards et al., 2010; Hoop et al., 2016). Whilst initial applications focused on organic molecules and retinal structures, recent applications have used this technique to probe the side chain dihedral angles of amino acids (Rienstra et al., 2002b; Bajaj et al., 2009; Edwards et al., 2010; Hoop et al., 2016). Like the NCCN experiment, these HCCH measurements can be applied to uniformly ^13^C labeled residues and polypeptides. In contrast to backbone torsion angles, the side chain angles are (at this time) not accessible via the kind of chemical shift analysis applied to polypeptide backbones (e.g., TALOS; Figure 2).

### Polyglutamine Amyloid Steric Zippers

One recent application of the HCCH experiment was also in our own work on aggregated polyglutamine (polyQ) proteins. As noted above, these protein aggregates are hard to study by distance measurements alone, requiring the application of dihedral angle measurements for structure determination. The glutamine residues feature an extended aliphatic side chain, with two methylene (CH_2_) groups for the Cβ and Cγ atoms. This permitted the use of HC-CH dihedral angle measurements of the χ_1_ and χ_2_ angles of these amino acids. Figure 4B shows how the χ_2_ angles of 60 and 180° give clearly different HCCH dephasing curves, with the dephasing at the mid-point (1/2 rotor period) varying with the χ_2_ angle (Figure 4C). For a practical experiment, measuring up to this mid-point is sufficient, as shown in Figures 4D,E. These results identified the χ_2_ angle for the glutamine residues in polyQ amyloid to be near 180°, in contradiction to certain prior structural models based on (low-resolution) X-ray diffraction (Sharma et al., 2005; Hoop et al., 2016). Similarly, the same experiments can be used to probe the χ_1_ angle (Figure 4F), adding further structural constraints on the polyQ amyloid core structure (Figure 4G). As summarized in Figure 4H, one unfortunate aspect of the χ_1_ measurements is that they were able to *exclude* various conformations, but did not result in a completely unambiguous single angle value. As noted above, this is not uncommon for dihedral angle measurements, which can provide a unique solution in some cases (χ_2_ ∼ 180°) but only partially constrain the angle in other cases. The model in Figure 4G represents a visualization of the best-fit backbone and side chain angle results. This model shows how the two thus-obtained conformers (β-strand types a and b) are conformationally compatible, and explain the co-assembly of the polyQ amyloid core. Notably, prior structural models derived from other techniques were not consistent with the obtained ssNMR results (Hoop et al., 2016).

### Receptor-Substrate Interactions

**FIGURE 5:**
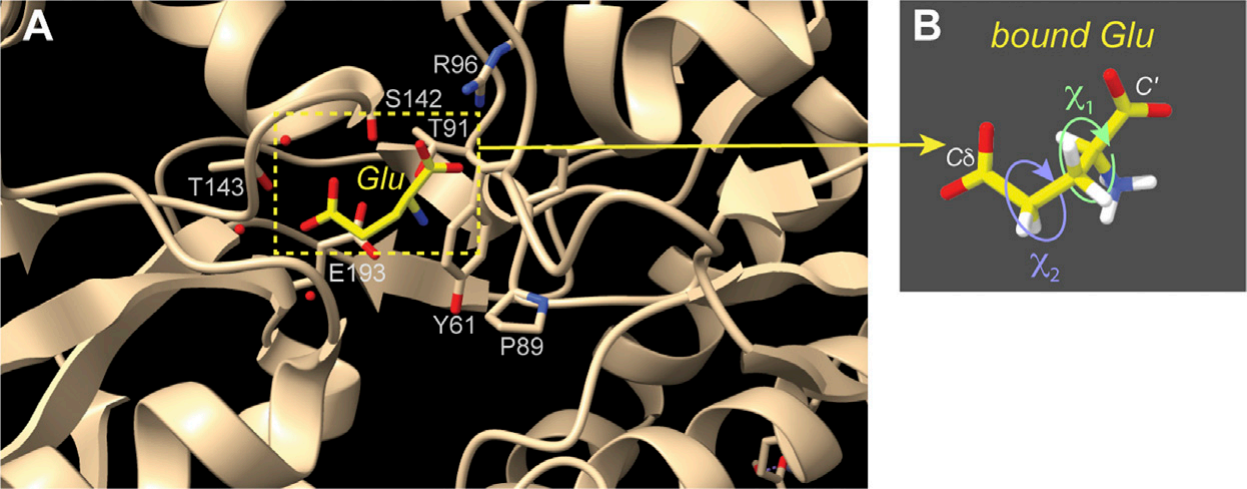
Receptor-bound substrate studied by torsion angle ssNMR measurements. **(A)** This image shows the structure of the receptor-bound Glu amino acid, based on X-ray crystallography (PDB 1FTJ) (Armstrong and Gouaux, 2000). Selected residues surrounding the bound substrate are indicated. **(B)** HCCH ssNMR analysis of χ_1_ and χ_2_ side chain angles enabled the measurement of the Glu residue structure while it was bound to the crystalline unlabeled protein (Edwards et al., 2010). This figure was prepared with UCSF ChimeraX (Pettersen et al., 2021).

Another notable use of the HCCH experiments is a nice study of a small molecule substrate (glutamate) bound to a receptor protein, which predated our work on polyQ (Edwards et al., 2010). An isotopically labeled substrate was bound to the ionotropic glutamate receptor 2, which was itself unlabeled (Figure 5). Two torsion angles, defining *χ*_1_ and *χ*_2_ of the Glu (Figure 5B), were measured with HCCH-type experiments. When combined with REDOR-based distance constraints, these data defined the conformation of the receptor-bound substrate. The reliability of the method was validated by comparison to known crystal structures (Figure 5A), with the proof-of-principle ssNMR study being applied to the crystalline receptor-substrate complex, in which the glutamate substrate is uniformly labeled. The HCCH measurements represented analogous experiments to our own subsequent studies of the polyQ amyloid structure (Figure 4). The individual dihedral angle measurements were again consistent with multiple distinct solutions, but by combining the different data with a REDOR-based distance measurement, a unique structural solution was obtained. The obtained conformation matched X-ray based structures previously determined, as illustrated in Figure 5 (Armstrong and Gouaux, 2000).

## Back to the Backbone–Alternative Implementations

**FIGURE 6:**
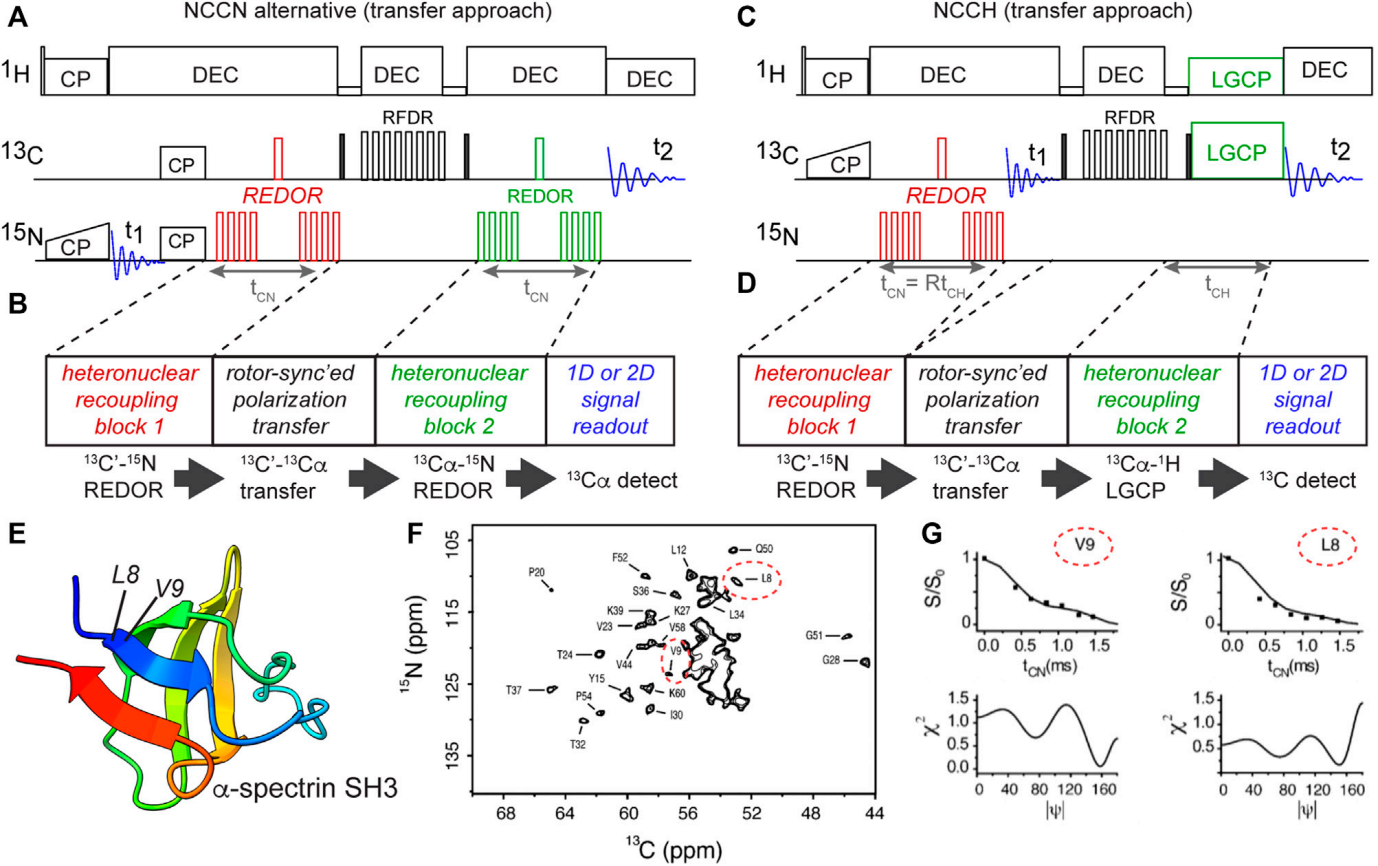
Transfer-based torsion angle measurements of backbone angle ψ. **(A,B)** NCCN pulse sequence implemented without ^13^C DQ generation, using rotor-synchronized ^13^C-^13^C transfer instead (Ladizhansky et al., 2003). Carbonyl (C′) polarization is created via a double CP scheme, followed by REDOR-based ^13^C-^15^N recoupling (time t_CN_). The residual ^13^C′ signal is transferred to neighboring ^13^Cα via a short RFDR block, after which the ^13^Cα-^15^N recoupling is done (also for t_CN_). Finally, the remaining ^13^C signal (as a function of t_CN_) is recorded in a series of 1D or 2D N(CO)CA spectra **(F)**. **(C,D)** NCCH-type measurements of the ψ backbone angle based on the same design principle (Ladizhansky et al., 2002). Here, ^13^C-^1^H dipolar recoupling occurred during the LGCP period. Note that here the REDOR time (t_CN_) and LGCP time (t_CH_) are both incremented in parallel. Panels B and D illustrate the generic design of these experiments, with color-coding of the dipolar recoupling blocks. **(E)** Structure of α-spectrin SH3, with residues L8 and V9 in the first β-strand marked (Musacchio et al., 1992), prepared with UCSF ChimeraX (Pettersen et al., 2021). **(F)** 2D N(CO)CA spectrum for uniformly labeled α-spectrin SH3, showing peaks for ^13^Cα_i_-^15^N_i+1_ correlations between residues i and i+1. Peaks for the Cα of residues L8 and V9 are indicated with red ovals. **(G)** Example N(CO)CA NCCN data curves for L8 and V9, along with matching values of the ψ angle. Panels **(F,G)** are adapted from (Ladizhansky et al., 2003) with permission, copyright American Chemical Society 2003.

In our hands, the abovementioned DQ-based approach is particularly powerful and robust. However, also alternative implementations of analogous torsion angle measurements have been demonstrated. This is illustrated via a different implementation of the NCCN-type measurement (Figures 6A,B) (Ladizhansky et al., 2003). This experiment follows the general approach shown in panel B: having two distinct recoupling blocks sandwiching a polarization transfer block. A key aspect of the pulse sequence is that each of these blocks should ideally be deployed in a rotor-synchronized (and/or constant-time) manner, such that individual molecules (crystallites) are recoupled at the same orientation relative to the magnetic field. This method has the benefit that it can be quite flexibly deployed, for example in the form of the HCCN experiment shown in Figures 6C,D (Ladizhansky et al., 2002). This HCCN experiment can be used to determine the ψ backbone angle, especially in α-helical secondary structures, thus complementing the NCCN experiment in terms of its applicability.

An illustration of the application of the abovementioned NCCN experiment from Figure 6A is shown in Figures 6E,F, from a study on crystalline uniformly ^13^C,^15^N-labeled α-spectrin protein (Figure 6E) (Ladizhansky et al., 2003). Integrating peaks in a series of 2D N(CO)CA spectra (Figure 6F), the REDOR dephasing curves for individual residues were measured. The β-sheet residues 8 and 9 are marked in the spectrum, with their NCCN curves shown in Figure 6G. Once again, multiple minima can be observed, e.g. for residue L8.

**FIGURE 7:**
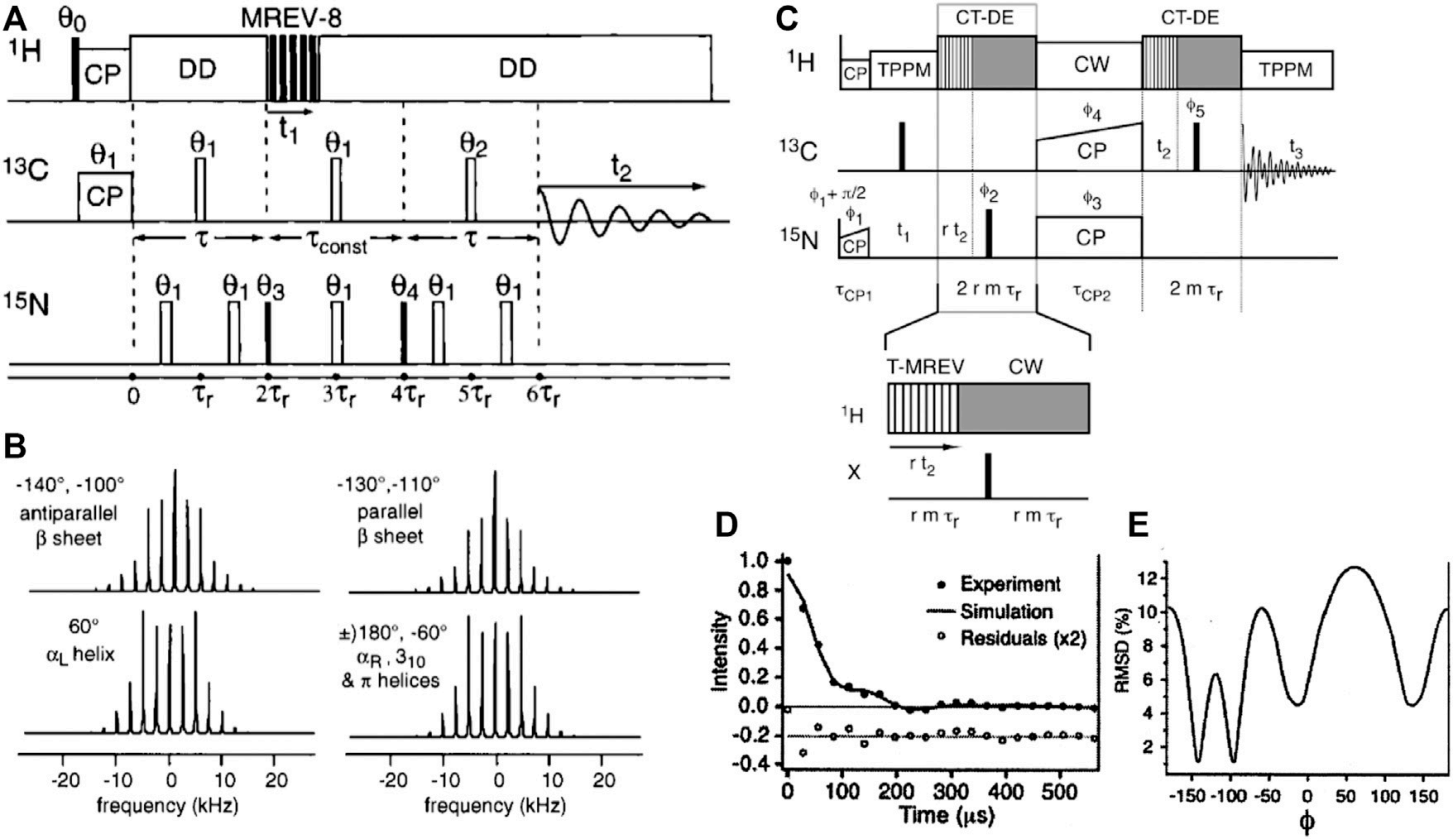
HCNH measurement of the ϕ backbone angle. **(A)** Pulse sequence for the HCNH experiment, where MREV-8 is used to achieve ^1^H-^13^C/^15^N dipolar recoupling of ^13^C-^15^N correlations. **(B)** Example dipolar lineshapes for four different ϕ angles, based on simulations. Note that one lineshape often represents two distinct ϕ angles, as indicated. The figure also lists the matching secondary structures, showing that each type of secondary structure has a characteristic spectral lineshape. **(C)** Pulse sequence of a HCNH experiment based on a transfer-like implementation similar to those shown in Figure 6. **(D,E)** Representative results showing how also this variant points to multiple minima. Adapted with permission from **(A,B)** ref. (Hong et al., 1997a) and **(C–E)** (Rienstra et al., 2002a), Copyright 1997 and 2002 American Chemical Society.

To measure the *ϕ* backbone angle one can use the HNCH ssNMR experiment (Hong et al., 1997a, Hong et al., 1997b; Hong, 1999; Rienstra et al., 2002a). Also this experiment can be implemented in different ways (Figure 7), either via coherence generation or a rotor-synchronized transfer approach (Rienstra et al., 2002a). The corresponding pulse sequences are illustrated in Figure 7, along with selected results from the literature. Once more these data illustrate how a single torsion angle measurement commonly is consistent with multiple best-fit minima. As noted above, this ambiguity can be resolved by performing multiple different torsion angle measurements, integration with chemical-shift-based analysis, consideration of accessible Ramachandran/rotamer space, and the measurement of relevant distance measurements. The reader is referred to prior review articles for a more in-depth discussion of these techniques (Hong and Wi, 2006; Ladizhansky, 2009).

## Using CSA Tensors for Measuring ω and More

**FIGURE 8:**
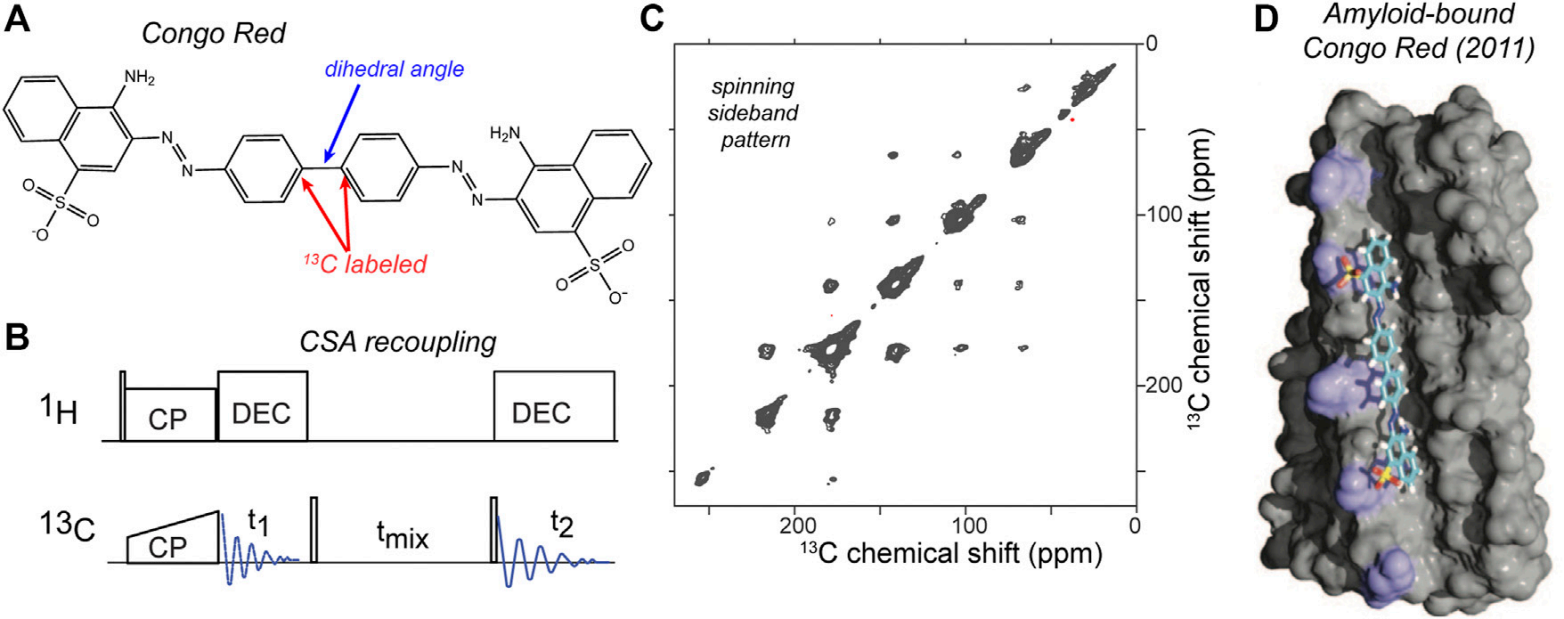
CSA-recoupling with selective labeling: amyloid dye Congo Red. **(A)** Chemical structure of congo red, which is widely used to detect amyloid structures. For the CSA recoupling experiments the 1 and 1′ carbons are ^13^C labelled (marked red), in order to measure the marked dihedral angle (blue). **(B)** Pulse sequence used for the CSA recoupling experiment used by (Gowda et al., 2017) to measure the dihedral angle marked in **(A)**. **(C)** 2D ^13^C-^13^C tensor correlation spectrum at 8 kHz MAS on a 850-MHz (^1^H) spectrometer, using the indicated pulse sequence with a 50-ms rotor-synchronized ^13^C-^13^C transfer time. The sample contained HET-s amyloid fibrils with the selectively labeled congo red bound. For more details see ref. (Gowda et al., 2017). **(D)** Structural model of amyloid-bound congo red from prior ssNMR studies by the same groups, adapted with permission from ref. (Schütz et al., 2011).

The most intuitive types of dihedral angle measurements are arguably those in which one recouples dipolar interactions that align nicely with chemical bonds, as discussed above. However, also the anisotropy of the chemical shift (CSA) can be deployed to good effect, either by recoupling two CSAs to each other, or by combining CSA recoupling with dipolar recoupling. Examples of these approaches were also introduced in the late 1990s (Ishii et al., 1996; Weliky and Tycko, 1996; Bower et al., 1999). The experiment in its simplest form involved simply slow MAS along with ^13^C-^13^C mixing, but this depended on ^13^CO-only labeling (Weliky and Tycko, 1996). It would not work in this form for a uniformly ^13^C labeled sample. An interesting recent application of this experiment was used to examine the conformation of the amyloid-binding fluorescent dye congo red, in its fibril-bound state (Figure 8). The CSA-based torsion angle measurement determined the central bond in the amyloid-bound dye, complementing other structural studies on the dye-fibril interactions by the same research groups (Schütz et al., 2011; Gowda et al., 2017). In this case, site-selective ^13^C labeling of the congo red was used to label two aromatic carbons. These aromatic sites have large ^13^C CSAs, with a well-understood orientation of the CSA tensor relative to the molecular frame. Thus, by determining the relative orientations of the two CSA tensors, one can determine the rotational angle marked in Figure 8A. Free rotation around this bond causes the unbound dye to display low fluorescence. Upon binding to amyloid fiber surfaces, the immobilization of the dye boost the fluorescence and permits thereby the selective detection of amyloid-like structures (Schütz et al., 2011; Yakupova et al., 2019). The experiment was performed with a fairly straightforward pulse sequence (Figure 8B), that allowed the measurement of a 2D ^13^C-^13^C spectrum that at slow MAS rates (here 8 kHz) showed a spinning side band pattern as illustrated in Figure 8C. Analysis of the peak pattern allowed the determination of the abovementioned torsion angle, which could then be used to refine the structural model of the congo red bound to the fiber surface grooves (Figure 8D). This represents a nice example of how one can target a torsion angle measurement to a particular (biological) question.

**FIGURE 9:**
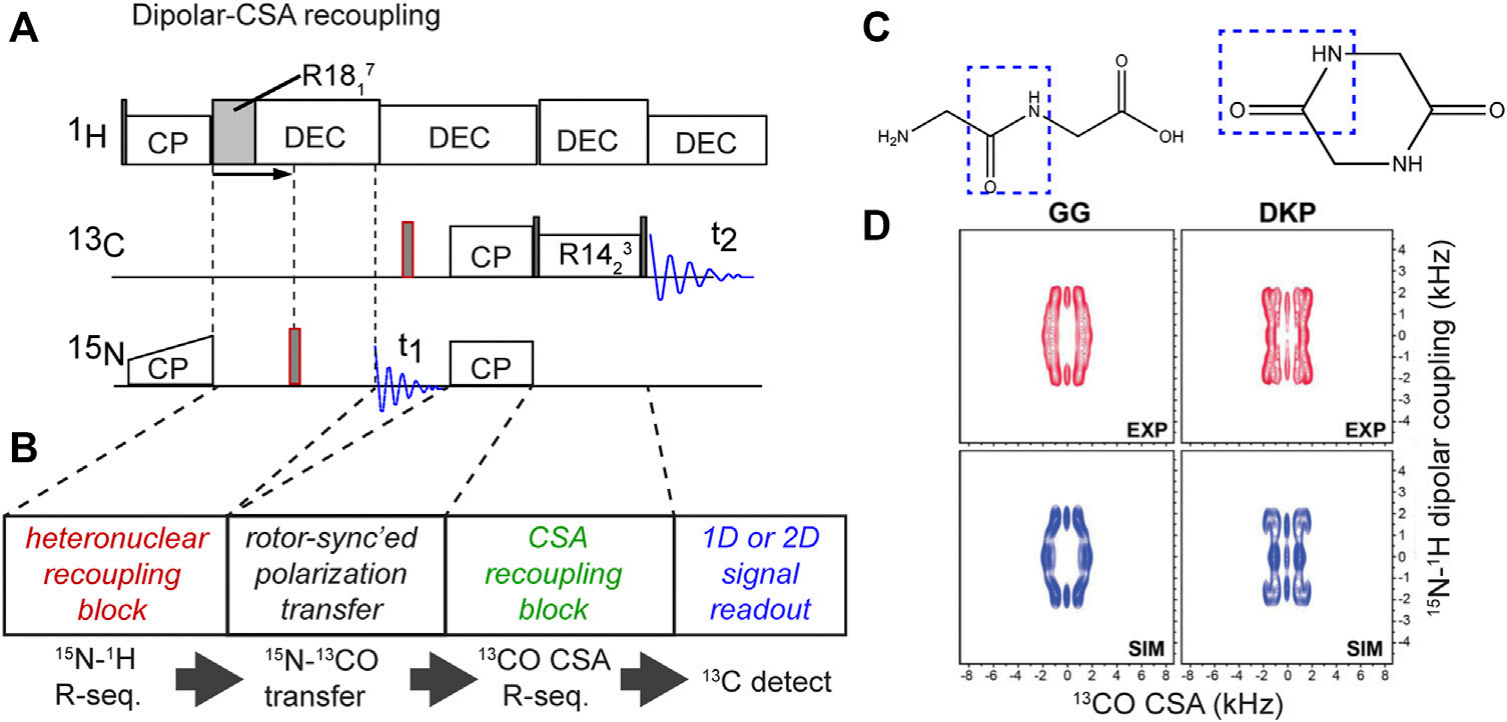
Hybrid CSA/dipolar recoupling experiment. **(A)** Pulse sequence and **(B)** its building blocks. **(C)** Chemical structures of test molecules with different molecular configurations mimicking the cis or trans peptide bond angle ω. **(D)** 2D CSA-dipolar correlation spectra for the two reference compounds representing the distinct ω angles. Panel (D) was adapted with permission from ref. (Mukhopadhyay et al., 2018).

Moreover, hybrid methods can also determine the relative orientations of a CSA tensor and a dipolar coupling (Ishii et al., 1996; Fujiwara et al., 1997; Hong et al., 1998; Chan and Tycko, 2003; Hou et al., 2010; Mukhopadhyay et al., 2018). In these MAS ssNMR experiments, different kinds of pulse sequence elements are used to actively recouple the CSA under medium/fast MAS (>10 kHz), unlike the slow-MAS (<10 kHz) CSA measurement mentioned above. For example, recent studies have used either ROCSA and R-sequence-based CSA recoupling techniques (Chan and Tycko, 2003; Hou et al., 2010; Mukhopadhyay et al., 2018). This approach also makes it feasible to apply these experiments without selective labeling strategies, enabling studies of fully ^13^C-labeled proteins. Figure 9 shows a recent example in which R-sequences were used for both CSA recoupling and dipolar recoupling, in order to enable measurement of the peptide bond angle ω (Mukhopadhyay et al., 2018). The figure shows the implementation and results for two model compounds (Figure 9C), but the original paper includes application to crystalline and fibrillar protein samples as well. This experiment and figure also illustrate a few relevant concepts, common to dihedral angle measurements. Figure 9D shows a 2D spectrum in which none of the axes show the (isotropic) chemical shift, but rather the anisotropic parameters: the ^13^C CSA and the ^1^H-^15^N dipolar coupling strength. Prior work has proposed the term Relayed Anisotropy Correlation spectra (RACO) (Ishii et al., 1996). In this experiment, these CSA and dipolar recoupling periods are both independently incremented, resulting in these typical 2D data. This figure illustrates how both of the (recoupling) time domain periods can be processed (Fourier transformed) in order to obtain a dipolar or CSA-based 2D lineshape. Although information-rich, this 2D approach does take considerable time. In line with prior work, this paper (Mukhopadhyay et al., 2018) discussed and demonstrated an “accordion” approach in which multiple distinct recoupling periods are incremented in synchrony. This yields a faster “1D” experiment that is must more time efficient than the 2D RACO spectra on uniformly labeled test compounds in Figure 9D. For more details, and the application to uniformly ^13^C,^15^N-labeled proteins, the reader is referred to the original work (Mukhopadhyay et al., 2018).

## Longer-Range Vector Angle Measurements

Most of the above examples are based on recoupling anisotropic interactions of neighboring (directly-bonded) atoms. However, useful angular constraints can also be derived from non-local interactions, involving atoms that are not directly bonded. Some CSA-CSA recoupling experiments fall into this category, but there are also dipolar based variants. This includes the recoupling of N-H dipolar vectors for ^15^N nuclei in neighboring amino acids, in HNNH-type experiments (Reif et al., 2000; Rienstra et al., 2002a, Rienstra et al., 2002b; Franks et al., 2006, Franks et al., 2008). The utility of such vector angle (VEAN) constraints was nicely demonstrated in the high-resolution structure determination of the model protein GB1 (Franks et al., 2006, Franks et al., 2008; Wylie et al., 2011). The obtained N-H, N-H VEAN depends on several standard protein dihedral angles, as well as bond lengths and bond angles. To use these constraints, the authors directly incorporated the VEAN angle into the structure calculation routine, rather than attempt to back-calculate the individual *ϕ*/ψ/*χ* angles. It is worth noting that these “remote” angle constraints can also take other forms, outside the HNNH variant, with any orientational constraint being potential valuable for structure determination. For example, the relative orientation (or projection angle θ) between (N-H)_i+1_ and (Cα-H)_i_ have been used to constrain amyloid structure of a fragment of the transthyretin protein (Jaroniec et al., 2004).

## Other ssNMR Probes of Dihedral Angles

### Distance-Based Constraints

Although not a focus of the current review, it is worth noting that many ssNMR distance measurements directly or indirectly constrain dihedral angles. In some cases, specific ssNMR experiments were designed with the explicit goal to determine dihedral angles via precise measurements of specific internuclear distances (Sinha and Hong, 2003; Wi and Spano, 2011; Hu et al., 2012; Pope et al., 2018). This includes for instance the so-called BARE (Backbone Recoupling) experiments that measure the distances between backbone nitrogens and carbonyls, with implications for the intervening backbone torsion angles (Hu et al., 2012).

### Isotropic Chemical Shifts as Torsion Angle Constraints

From the above it is clear that there is an impressive library of ssNMR torsion angle measurements, many of which were developed and demonstrated in the late 1990s and early 2000s. Nonentheless, since then many ssNMR protein structures were determined without use of these types of constraints. Instead, most structural ssNMR studies currently deploy isotropic chemical shifts to estimate the residue-specific backbone torsion angles. This is facilitated by the observation that backbone chemical shifts (along with the Cβ shift) are reliable indicators of local secondary structure, and can even be used for quantitative determinations of backbone dihedral angles (Cornilescu et al., 1999; Shen and Bax, 2007; Shen et al., 2009). Although developed for (and from) solution NMR protein structures, these algorithms have been shown to be similarly effective for ssNMR chemical shifts. These results have been sufficiently reliable that several papers deploying explicit torsion angle measurements note the high degree of consistency between the two approaches (Jaroniec et al., 2004; van der Wel et al., 2010). That said, both methods have their strengths and weaknesses, and can used as complementary tools (van der Wel et al., 2010; Hoop et al., 2016). For example, chemical shift-based estimates can be used to resolve ambiguities inherent in direct dihedral angle measurements (and *vice versa*).

## Prospects for Torsion Angle Applications

These recent studies give a chance to consider the question of when and why to deploy torsion angle measurements. These experiments are in principle powerful, and they been used in recent years, but clearly not as widely as other structural ssNMR measurements. There are several reasons of this. One reason is that protein chemical shifts themselves give a lot of insight into the backbone torsion angles (i.e. secondary structure), with programs like TALOS allowing for a semi-quantitative determination of the backbone torsion angles (Shen et al., 2009; van der Wel et al., 2010). Most recent ssNMR-based structures are based on combinations of distance measurements along with such chemical-shift-based backbone angles. Although (typically) neither the distances nor the angles are extremely precise, their combination in sufficient numbers can yield good atomic structures of proteins (Loquet et al., 2008). Dihedral angle measurements may be used to improve the resolution of a structure derived from a combination of distance constraints and chemical shift information. This has been demonstrated for example in work by the Rienstra group on crystals of the model protein GB1 (Franks et al., 2008; Wylie et al., 2011). This enables higher resolution structures than otherwise accessible, but the question may arise whether the improvement in the structure quality warrants the amount of work (NMR time, but also data analysis). One possible approach would be to deploy these experiments in a targeted fashion: to enhance our understanding of especially important parts of protein structures, such as enzyme active sites, ion channel selectivity pores and similar (van der Wel et al., 2009; Caulkins et al., 2014; Wylie et al., 2014). Still, the implementation, execution, and interpretation of torsion angle measurements can be challenging, more so due to more limited prior experience with these methods in the overall ssNMR community (compared to canonical distance measurements). Thus, it may seem unclear when one would deploy dihedral angle measurements. We will examine some considerations or conditions that favor their use.

### Extensive Intermolecular Interactions

Distance-based ssNMR structure determination borrows heavily from methods perfected in solution NMR structural biology. In dissolved or crystalline globular proteins, or in membrane-associated proteins, one can assume that most NMR-detected distance constraints reflect interactions within an individual protein (Loquet et al., 2008). However, this is not always a safe assumption, for example when studying amyloid fibrils in which the predominant residue-residue interactions may be inter- rather than intra-molecular. SSNMR studies of amyloid structures have addressed this by diluting labeled monomers in an excess of unlabeled monomers, prior to the assembly process. This approach allows for the suppression of intermolecular interactions among (labeled) atoms, thus revealing intramolecular interactions that define the monomer structure within the fibril. Unfortunately, it also implies a drastic loss of sensitivity, as the sample is now only partly filled with “visible” labeled monomers. Torsion angle measurements are designed to probe the very local environment of the (bond) angle of interest. This means that they deploy relatively modest dipolar recoupling times and are largely insensitive to the presence or absence of intermolecular interactions, and that they can always be used to probe the monomer structure even in fully labeled samples. Aside from the already mentioned signal-to-noise benefits, this may also be important for (biological) assemblies that are difficult or impossible to (re)assemble *in vitro* from monomers, and thus are either fully labeled or fully unlabeled.

### Assemblies With Inherent or Internal Polymorphism

Another challenge faced in our recent studies of amyloid fibrils is that some fibrils contain the same monomer in two or three distinct configurations or conformations, as part of a complex or composite fiber architecture (van der Wel et al., 2010; Lewandowski et al., 2011; Hoop et al., 2016). This means that any single atom (or residue) yields multiple signals, which further complicates distance-based structural measurements (E.g., Figures 3, 4 show examples). Extensive signal overlap results that cannot be resolved by site-specific isotope labeling or spin-system-based spectroscopic editing. Moreover, the effective signal to noise is decreased, as you are effectively studying a system that behaves as if it is twice or thrice as large (in terms of signal to noise). Yet, unlike a larger protein sequence, this challenge cannot be resolved by residue-specific labeling, truncation of the sequence, mutations, or segmental labeling. In such a case, distance measurements become less powerful, and torsion angle measurements can become an essential tool.

### Cases Where Chemical Shift Analysis Falls Short

It was noted above that backbone dihedral angles can be probed via the chemical shift assignments of proteins, with the results sometimes being not much “worse” than much more time-consuming torsion angle measurements (Figure 2F). However, even if the detection of extended β-strands and α-helices is quite reliable, some non-standard motifs can be more challenging. In such cases chemical shift-based analysis alone may fail to resolve a reliable backbone conformation for a combination of observed shift values (see e.g., Figure 2J). Thus, it may be helpful to deploy targeted torsion angle measurements in such cases (Franks et al., 2008; van der Wel et al., 2010). Moreover, chemical shift analysis is limited to protein backbones, while ssNMR torsion angle measurements can be applied to side chains and non-protein biomolecules.

### Repetitive Sequences and Structures

Our work on polyQ amyloid structure illustrates one interesting class of proteins where distance constraints fail to provide a complete answer. Highly repetitive sequences such as the polyQ proteins render distance constraints more difficult to obtain, or at least interpret. Biology features many repetitive protein sequences. Protein aggregation diseases often feature proteins with low complexity sequences, which are sometimes defined as prion-like sequence elements (Franzmann and Alberti, 2019). Besides several distinct polyQ disease proteins and highly sequence-repetitive prions, much interest extends also to the repetitive dipeptide-repeats associated with ALS disease (Odeh and Shorter, 2020; Schmitz et al., 2021). The aggregated and phase-separated states of these proteins remain as yet poorly understood, with studies by ssNMR likely being important to understanding their structure, dynamics and phase behavior. However, repetitive sequences go well beyond amyloidogenic proteins. A different example is collagen, which is an essential component of the extracellular matrix (ECM), where it helps define the structural characteristics of tissues. The (mechanical) properties of the ECM are of substantial biological interest, for instance in the context of cancer research and treatments (Venning et al., 2015). Already a topic of significant ssNMR studies (Goldberga et al., 2018), collagen’s repetitive structure yields highly challenging spectra with highly overlapping signals, akin to the polyQ case study above. Dihedral angle measurements may be similarly useful for nonetheless probing the local structure in atomic detail. Similarly, the silk proteins produced by spiders and other animals are highly repetitive in sequence and have interesting structural properties that have been studied by ssNMR (van Beek and Meier, 2006; Holland et al., 2008). Also here dihedral angle measurements can be useful to probe their structures and structural transitions (van Beek and Meier, 2006).

### Applications Beyond Polypeptides

Mostly we have focused on the study of protein samples. However, the use of dihedral angle measurements is also of interest for the study of other biomolecules (or non-biological samples). Besides repetitive protein structures, we may also consider applications to non-protein macromolecules of biological, biomedical and bioengineering interest. For instance (high-molecular weight) polysaccharides, RNA and DNA are increasingly studied by ssNMR, but present new challenges in terms of structure determination (Marchanka et al., 2015; El Hariri El Nokab and van der Wel, 2020).

However, also in small molecules (or short peptides), it can be difficult to gain sufficient information from distance constraints alone. In such a case, dihedral angle measurements can play an important role. In the context of peptides, this has now been well demonstrated (Rienstra et al., 2002b; Jaroniec et al., 2004; Bajaj et al., 2009; Barnes et al., 2009; van der Wel et al., 2010). Nice examples can also be found in the earlier literature, for example in studies of the retinal of the membrane protein rhodopsin (Feng et al., 1997b). The recent literature offers several other interesting case studies, as we have already seen above, in which torsion angle measurements probe the structure of small molecules bound to proteins, rather than the protein itself. This includes the example of a small molecule substrate bound to a receptor protein (Figure 5) (Edwards et al., 2010) and the amyloid-specific fluorescent dye congo red bound to HET-s fibrils (Gowda et al., 2017). It is likely that further applications like this can be expected in future work on, e.g., drug-protein, substrate-enzyme and other such interactions.

## Technical Challenges and Opportunities

As with any (ssNMR) experiment, the torsion angle measurements offer both unique strengths and also specific challenges. This section notes a few specific challenges, but follows this with perspectives on how they can be overcome (partly with the help of modern MAS ssNMR equipment). Like with other structural ssNMR studies reliant on anisotropic interactions, any dynamics in the molecular system can interfere with the execution and/or analysis of torsion angles. Since dynamics modulate both dipolar interactions and CSA tensors, this would naturally cause problems. These dynamics can be important and relevant, since biological contexts often require dynamics (e.g., in enzymes or ion channels) to achieve proper function. On the one hand, it may be possible to account for certain types of (anisotropic) motion in the data analysis, as exemplified in prior studies that characterized such motion by ssNMR (Hu et al., 2010; Li and Hong, 2011). Another workaround could be the use of low-temperature experiments, which are increasingly accessible with the enhanced availability of low-temperature dynamic nuclear polarization (DNP) equipment (Lilly Thankamony et al., 2017), and can permit the execution of dihedral angle measurements at reduced temperatures where molecular motion is suppressed (Bajaj et al., 2009).

One challenge independent of motion is the inherently low sensitivity of the experiments. Whilst applications to crystalline peptides have been quite effective and convincing, applications to more complex systems are fairly demanding. As already discussed above, a combination of factors contribute to this challenge. On the one hand, the nature of the pulse sequences is such the signals are purposely decreased, and we monitor the differential degree of dephasing to distinguish the differences in structure. Crucially, for several types of measurements certain ranges of angles are close together in terms of their dephasing curves. This places significant demands on the signal to noise. The good news is the techniques and equipment available for MAS ssNMR have greatly improved since the dihedral angle measurements were first developed. On the one hand, we already discussed DNP for its low temperature capabilities. This technique also offers substantial signal enhancements, which even permit applications to natural abundance proteins and materials within reach. Notably, recent DNP studies of unlabeled proteins and other organic (bio)molecules (Märker et al., 2017; Smith et al., 2018) already depend heavily on the kinds of DQ experiments at the heart of several types of dihedral angle measurements, as reviewed above.

In addition to DNP technology, we also see the gains in the application of high field ssNMR and especially also (^1^H-detected) fast MAS (>60 kHz). Most of the example data discussed in this review article were obtained with MAS rates between 10 and 15 kHz (reflecting typically 3.2 and 4 mm MAS rotor diameters). High MAS rates that exceed even 100 kHz are now available, permitting ^1^H detection and other new pulse sequences (Barbet-Massin et al., 2014; Zhang et al., 2017; Ji et al., 2021). This can enable new types of experiments and thereby new structural insights, often with improved sensitivity and time-savings. However at the same time, some traditional pulse sequence elements become difficult or impossible to implement, for instance due to the overly short rotor period lengths or the high RF power requirements. This for instance means that REDOR-based methods may not work, requiring the deployment of new schemes. This has opened up new developments of various sorts of MAS ssNMR experiments, such as tailored assignment and relaxation measurements (Barbet-Massin et al., 2014; Zhang et al., 2017; Ji et al., 2021), but the development of (new) dihedral angle experiments has lagged behind (Hou et al., 2010).

A final practical challenge relates to the implementation and analysis of torsion angle experiments. Fewer groups have hands-on experience with dihedral angle experiments, compared to the more widely used distance measurements. Moreover, the configuration and implementation of these experiments is arguably more involved than, e.g., a traditional DARR-based distance measurement. Also the interpretation of the obtained results is perhaps less intuitive than looking for the presence or absence of cross peaks in typical distance measurements. One helpful development is the availability of various efficient and flexible numerical simulation packages, which can facilitate both test simulations to better understand the use of these experiments, and can also enable efficient analysis and interpretation of obtained results (Bak et al., 2000; Veshtort and Griffin, 2006).

With all these technical and hardware enhancements, it seems likely that many new and useful dihedral angle measurements may be introduced in the future. And it also seems plausible that existing techniques may find wider adoption and application to suitable systems, whether biological or non-biological in nature.

## Discussion

In this article we have examined several recent applications and methodologies of ssNMR dihedral angle measurements, focusing on those techniques based on the recoupling of anisotropic dipolar and/or CSA tensors. With the advent of highly productive distance and shift-based structure determination techniques for ssNMR-based structural biology, these direct dihedral angle measurements have been a bit left outside the mainstream. Yet, we have seen how they can be powerful and essential for tackling various biologically important questions, ranging from amyloid fiber structure determination to receptor-substrate interactions. It is furthermore anticipated that there is significant room for enhancing the utility of these techniques, as future studies will surely integrate these techniques with modern MAS ssNMR techniques such as ^1^H-detected fast MAS and DNP. Thus, we foresee an expansion of the role of these methods toward broader application in the ssNMR community, with valuable roles in studies of biological and non-biological systems, both with and without stable-isotope labeling.
