# Correction: Dihedral angle measurements for structure determination by biomolecular solid-state NMR spectroscopy

**DOI:** 10.3389/fmolb.2025.1716334

**Published:** 2025-11-07

**Authors:** Patrick C. A. van der Wel

**Affiliations:** Solid-state NMR Group, Zernike Institute for Advanced Materials, University of Groningen, Groningen, Netherlands

**Keywords:** solid-state NMR, structural biology, amyloid, polyglutamine, protein structures

There was a mistake in the caption of **Figure 6** as published. The caption erroneously stated “(C,D) NCCH-type measurements of the φ backbone angle based on the same design principle (Ladizhansky et al., 2002)”. This text should have been referring to the ψ backbone angle. The corrected caption of **Figure 6** appears below.

“FIGURE 6 Transfer-based torsion angle measurements of backbone angle ψ. **(A,B)** NCCN pulse sequence implemented without ^13^C DQ generation, using rotor-synchronized ^13^C-^13^C transfer instead (Ladizhansky et al., 2003). Carbonyl (C′) polarization is created via a double CP scheme, followed by REDOR-based ^13^C-^15^N recoupling (time t_CN_). The residual ^13^C′ signal is transferred to neighboring ^13^Cα via a short RFDR block, after which the ^13^Cα-^15^N recoupling is done (also for t_CN_). Finally, the remaining ^13^C signal (as a function of t_CN_) is recorded in a series of 1D or 2D N(CO)CA spectra **(F)**. **(C,D)** NCCH-type measurements of the ψ backbone angle based on the same design principle (Ladizhansky et al., 2002). Here, ^13^C-^1^H dipolar recoupling occurred during the LGCP period. Note that here the REDOR time (t_CN_) and LGCP time (t_CH_) are both incremented in parallel. Panels B and D illustrate the generic design of these experiments, with color-coding of the dipolar recoupling blocks. **(E)** Structure of α-spectrin SH3, with residues L8 and V9 in the first β-strand marked (Musacchio et al., 1992), prepared with UCSF ChimeraX (Pettersen et al., 2021). **(F)** 2D N(CO)CA spectrum for uniformly labeled α-spectrin SH3, showing peaks for ^13^Cα_i_-^15^N_i+1_ correlations between residues i and i+1. Peaks for the Cα of residues L8 and V9 are indicated with red ovals. **(G)** Example N(CO)CA NCCN data curves for L8 and V9, along with matching values of the ψ angle. **(F,G)** are adapted from (Ladizhansky et al., 2003) with permission, copyright American Chemical Society 2003.”

The original article has been updated.

